# Cas12a and Lateral Flow Strip-Based Test for Rapid and Ultrasensitive Detection of Spinal Muscular Atrophy

**DOI:** 10.3390/bios11050154

**Published:** 2021-05-14

**Authors:** Chunhua Zhang, Zhuo Li, Miaomiao Chen, Zhiqing Hu, Lingqian Wu, Miaojin Zhou, Desheng Liang

**Affiliations:** Center for Medical Genetics & Hunan Key Laboratory of Medical Genetics, School of Life Sciences, Central South University, Changsha 410078, China; zhangchunhua@sklmg.edu.cn (C.Z.); lizhuo@sklmg.edu.cn (Z.L.); chenmiaomiao@sklmg.edu.cn (M.C.); huzhiqing@sklmg.edu.cn (Z.H.); wulingqian@sklmg.edu.cn (L.W.)

**Keywords:** Cas12a, lateral flow strip, detection, spinal muscular atrophy

## Abstract

Spinal muscular atrophy (SMA) is characterized by severe lethality and irreversible progression. Early diagnosis of SMA is of more practical significance with the emergence of effective therapy. However, existing techniques to identify SMA patients rely on cumbersome instruments, hindering their accessibility and application. An SMA-Cas12a-strip assay was developed with the integration of Cas12a-based nucleic acid detection, isothermal amplification, and lateral flow strip. The analytical performance of the assay was assessed with clinical samples. To explore its extensible utility, various specimens were tested. Validated with 168 clinical samples, the sensitivity and specificity of the SMA-Cas12a-strip assay were both 100%. The minimum detectable concentration of genomic DNA containing the target gene achieved 526 aM. The assay was compatible with specimens from several sources, and the turnaround time could be within 1.5 h. We developed a simple, cost-effective, and highly sensitive and specific assay to detect SMA patients. With little and field-portable equipment, the assay holds great promise in the detection of SMA patients, particularly in low-resource regions.

## 1. Introduction

Spinal muscular atrophy (SMA) is an autosomal recessive neurodegenerative disorder and is one of the most common genetic diseases leading to infant death, with a reported incidence of 1 in 6000–10,000 live births and a carrier frequency of 1 in 50 in the general population [1,2]. SMA is featured by the irreversible degeneration of motor neurons in the spinal cord, which leads to progressive muscle weakness and respiratory failure. Survival motor neuron 1 gene (*SMN1*) is the causative gene of SMA [3], and more than 95% of SMA patients have a homozygous deletion of the *SMN1* exon 7 [4,5]. Although the survival motor neuron 2 gene (*SMN2*) is highly homologous to *SMN1* in humans, *SMN2* cannot fully compensate for the loss of *SMN1* [6].

Despite a better understanding of the etiology and pathogenesis of SMA, it had been considered as an incurable disease until the emergence of Nusinersen, Zolgensma, and Risdiplam, which are efficacious to improve survival and motor function in clinical trials, and have been approved for clinical application [7,8,9,10]. Predictably, more therapeutic approaches will be available in the near future, but another issue of how to identify presymptomatic SMA patients will be more prominent. Only the adoption of treatment within an optimal therapeutic time window can maximize the benefit from therapy, so the early detection of SMA, for instance, through newborn screening (NBS), is pertinent and imperative [11,12].

Several techniques to detect SMA have been developed, such as multiplex ligation-dependent probe amplification (MLPA), quantitative PCR (qPCR), high-resolution melt profiling (HRM), denature high-performance liquid chromatography (DHPLC), and digital PCR (dPCR) [13,14,15,16,17,18]. However, these technical platforms rely on bulky instruments and trained personnel, hindering their application in developing countries or low-resource regions.

Novel nucleic acid detection strategies based on CRISPR/Cas systems (SHERLOCK, DETECTR, and HOLMES) have emerged and have a high sensitivity, high specificity, and low cost [19,20,21,22,23], but the potential utility of the CRISPR/Cas nucleic acid detection in detection for human genetic diseases needs further exploration.

With the integration of Cas12a-based nucleic acid detection, isothermal amplification, and a lateral flow strip, we aimed to establish a method with convenience and low costs to detect the homozygous deletion of *SMN1* exon 7 in SMA patients.

## 2. Materials and Methods

### 2.1. Source of Samples

This study was approved by the Ethics Committee of the School of Life Sciences, Central South University. Deidentified samples were obtained from Hunan Jiahui Genetics Hospital, and informed consent was obtained from all sample donors. The detailed information of all samples was summarized in Tables S1 and S2 (in the supplementary materials).

### 2.2. Primers and crRNAs

PCR primers SMN1 F1/R1 were designed with the routine principle, by which exon 7 of the human *SMN1* gene (NM_000344) and *SMN2* gene (NM_017411) can be amplified. Recombinase polymerase amplification (RPA) primers were designed following the guidance of TwistAmp DNA Amplification Kits (TwistDx, Cambridge, UK). RPA primers SMN1 F2/R2 can amplify both *SMN1* and *SMN2*. RPA primers SMN1 F3/R3, the 3′-end of which is located at the unique site c.835-44G and c.*3 + 100A of *SMN1*, theoretically could specifically amplify *SMN1* but not *SMN2*.

Cas12a is sensitive to mismatches between crRNA and target dsDNA, and especially cannot tolerate mismatches at the 5′-end region of crRNA [24,25], which is helpful for improving the specificity of recognizing the *SMN1* exon 7. crRNAs targeting the *SMN1* exon 7 were designed with the web tool CHOPCHOP [26]. Intrinsic single nucleotide variation (SNV) at c.840 was included at the 5′-end region of SMN1 crRNA-wt. Another mismatch besides c.840 was artificially introduced into SMN1 crRNA-mut to recognize the *SMN1* exon 7 more specifically.

In order to explore the extensibility of a Cas12a-based test, Duchenne muscular dystrophy (DMD) was selected as another target disease, and crRNAs and RPA primers targeting exon 10, exon 16, exon 45, and exon 50 of the dystrophin gene (*DMD*, NM_004006) were designed. Detailed sequence information of all premiers, crRNAs, and fluorescence probes was provided in Table S3.

### 2.3. DNA Amplification

Two DNA amplification methods (PCR and RPA) were used in this study. PCR was conducted as a routine procedure. RPA was conducted on a dry bath incubator with the TwistAmp Liquid Basic Kit (TwistDx). Fifty microliters of the RPA reaction system contained 25 μL of buffer, 9.2 μL of dNTP (10 μM), 5 μL of E-mix, 2.5 μL of the core reaction mix, 2.5 μL of MgOAc (280 mM), 2.4 μL of forward and reverse RPA primer, respectively (10 μM), and 1 μL of DNA template. The amplification condition of RPA was 40 °C for 40 min.

### 2.4. The SMA-Cas12a Assay with a Fluorescence Probe

The SMA-Cas12a assay was established with a fluorescence probe, and the fluorescence signal could be detected quantitatively, which was conducive to the optimization and validation of crRNAs. The 20 μL of the SMA-Cas12a assay system with a fluorescence probe contained 1 μL of Lba Cas12a (2 μM) (NEW ENGLAND BioLabs, NEB, Ipswich, MA, USA), 2 μL of NEBuffer 2.1 (NEB), 1 μL of crRNA (1 μM), 2 μL of PCR products, 0.8 μL of fluorescence probe (10 μM) and 13.2 μL of H_2_O. The reaction system was incubated at 37 °C for 40 min, and the fluorescence signal was measured every minute. To explore whether pre-amplification was necessary, a DNA sample with a concentration of 1000 ng/μL from a non-SMA individual and its PCR products were tested by the SMA-Cas12a assay with the fluorescence probe. The same DNA sample was diluted serially to determine the minimum concentration of DNA that could be detected by the SMA-Cas12a assay with the fluorescence probe. The recognition specificity was also compared between SMN1 crRNA-wt and SMN1 crRNA-mut.

### 2.5. Selecting Optimal RPA Primers

A DNA sample from a non-SMA individual was amplified by RPA with primer pairs of F2/R2 or F3/R3, respectively. RPA products could not be analyzed directly because of Carbowax (as an ingredient in the RPA kit), so RPA products were purified by the Cycle Pure Kit (Omega Bio-tek, Norcross, GA, USA). After purification, amplification products were analyzed by Sanger sequencing.

### 2.6. The SMA-Cas12a-Strip Assay with an FITC-Biotin-Labeled Probe

The volume of the Cas12a cleavage reaction system was 20 μL, containing 1 μL of Lba Cas12a (5 μM) (NEB), 2 μL of NEBuffer 2.1 (NEB), 1μL of crRNA (0.5 μM), 2 μL of RPA products, 1 μL of the FITC-Biotin-labeled probe (0.05 μM), and 14 μL of H_2_O. The reaction was performed at 37 °C for 5 min, 10 min, 15 min, and 20 min to determine the optimal incubation time. After incubation, the 20 μL of reaction volume was mixed with 100 μL of HybriDetect assay buffer (Milenia Biotec, Giessen, Germany) in a 1.5 mL tube. A lateral flow strip (Milenia) was placed into the tube uprightly at room temperature. After the control band (top in the strip) manifested the signal obviously and completely, the signal on the test band (low in the strip) could be visually observed by naked eyes and captured by the camera to record.

To determine the minimum detectable concentration of DNA, a DNA sample from a healthy donor was serially diluted into concentrations of 1000, 100, 10, 1, 0.1, and 0.01 ng/μL, and these samples were tested by the SMA-Cas12a-strip assay.

### 2.7. Evaluation of Analytical Performance of the SMA-Cas12a-Strip Assay

To evaluate sensitivity and specificity, 168 DNA samples were tested by the SMA-Cas12a-strip assay. The 168 samples had been tested by MLPA and qPCR previously to confirm the copy numbers of *SMN1* exon 7 and had been classified as being from SMA patients or non-SMA individuals. For SMA patients, those who tested positive by the SMA-Cas12a-strip assay were defined as true positive (TP), and those who tested negative were defined as false negative (FN). For non-SMA individuals, those who tested negative were defined as true negative (TN), and those who tested positive were defined as false positive (FP). TP, FP, TN, and FN were used to calculate sensitivity and specificity.

### 2.8. Exploring Extensible Utility

A convenient DNA extraction method was adopted to extract DNA from specimens of DBS, oral swabs, peripheral blood, hair follicles, and amniotic fluid with the RoomTemp Sample Lysis Kit (Vazyme Biotech, Nanjing, China). Briefly, 2 μL of peripheral blood or precipitation of amniotic fluid (about 3000 amniocytes) was added to 20 μL of lysis buffer, and this mixture was incubated at room temperature for 3 min and then added to 20 μL of stabilizing buffer. There were some alterations in the extracting procedure for specimens of DBS and hair follicles. Incubation occurred at 95 °C for 3 min, and the volume of the lysis buffer and stabilizing buffer was 50 μL. For oral swabs, samples were added with 400 μL of lysis buffer, incubated at 95 °C for 3 min, and were then added with 400 μL of stabilizing buffer. DNA samples from different sources were tested by the SMA-Cas12a-strip assay to explore its compatibility with these specimens.

DNA samples of 7 DMD patients and 5 non-DMD individuals were tested with the DMD-Cas12a-strip assay following the same protocols as those of the SMA-Cas12a-strip assay, except for the substitution of the corresponding RPA primers and crRNAs.

### 2.9. Statistical Analysis

Statistical analyses were performed using GraphPad Prism 8 (GraphPad Software), two-group data using a Student’s *t*-test, and multi-group data using one-way analysis of variance (ANOVA). *p* < 0.05 was considered statistically significant.

## 3. Results

### 3.1. Establishment of the SMA-Cas12a Assay with a Fluorescence Probe

**Figure 1:**
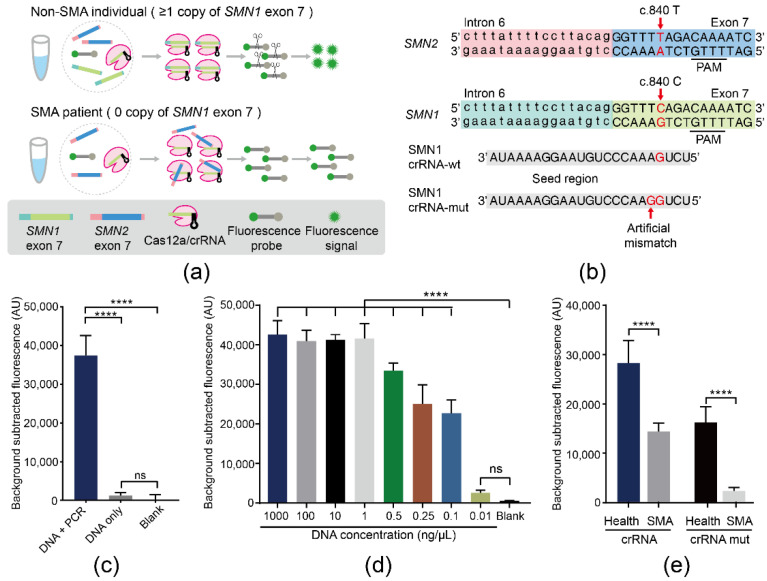
The SMA-Cas12a assay with a fluorescence probe. (**a**) In non-SMA individuals, *SMN1* exon 7 triggered Cas12a after being recognized by the Cas12a/crRNA complex. Activated Cas12a acquired collateral cleavage activity to nonspecifically degrade the vicinal fluorescence probe yielding fluorescence. However, SMA patients had no *SMN1* exon 7 to activate Cas12a and could not generate fluorescence. (**b**) Design of SMN1 crRNA-wt and SMN1 crRNA-mut. (**c**) Results of comparing the magnitude of fluorescence in three groups: DNA amplified by PCR, DNA without amplification, and the blank control. Data are means ± SD, (**** *p* < 0.0001, ns, *p* > 0.05). (**d**) The minimum detectable concentration of genomic DNA of the SMA-Cas12a assay with a fluorescence probe. Data are means ± SD, (**** *p* < 0.0001, ns, *p* > 0.05). (**e**) Results from comparing SMN1 crRNA-wt with SMN1 crRNA-mut in the capacity of discriminating SMA patients and normal individuals. Data are means ± SD, (**** *p* < 0.0001).

The principle of the SMA-Cas12a assay is illustrated in Figure 1a. Two crRNAs of SMN1 crRNA-wt and SMN1 crRNA-mut were designed and synthesized (Figure 1b). PCR products of the DNA sample from a non-SMA individual showed a significantly higher magnitude of fluorescence than the DNA sample without amplification. Without PCR, the DNA sample failed to generate an obvious fluorescence signal compared with the blank control. These results suggested that the pre-amplification was necessary for the assay (Figure 1c). Amplified by PCR, the minimum detectable concentration of DNA by the SMA-Cas12a assay with the fluorescence probe could reach 0.1 ng/μL (Figure 1d). Subsequently, pre-amplification was performed with RPA and primer pairs F2/R2. Guided by SMN1 crRNA-wt, the fluorescence of non-SMA individuals and SMA patients showed a 2.26-fold difference, but the nonspecific activation of Cas12a by *SMN2* was still present. In order to avoid cross-reaction of *SMN2*, an artificial mismatch adjacent to c.840 was introduced into SMN1 crRNA-mut. Guided by SMN1 crRNA-mut, the difference between non-SMA individuals and SMA patients reached 7.41-fold (Figure 1e).

### 3.2. Specific Amplification of SMN1 by RPA

**Figure 2:**
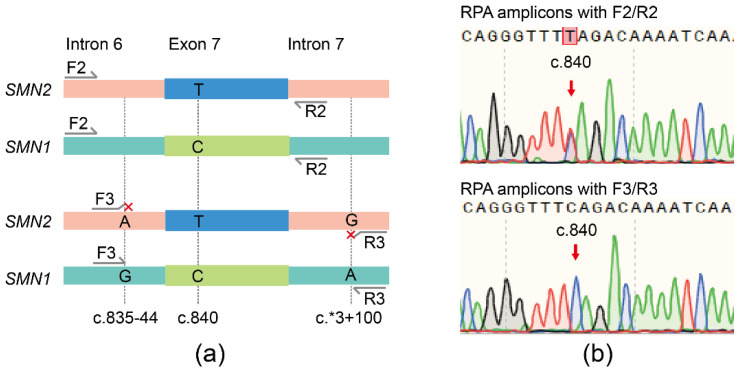
Diagram of RPA primers and sequencing results of RPA products. (**a**) Innate SNVs between *SMN1* and *SMN2* located at the 3′ end of primers F3/R3. (**b**) Sanger sequencing of RPA products amplified with the primers F2/R2 and F3/R3.

Although RPA and SMN1 crRNA were applicable, the specificity of the assay could be further improved through the optimization of primers. DNA samples from a non-SMA individual containing both *SMN1* and *SMN2* were amplified by RPA with primer pairs F2/R2 or F3/R3, respectively (Figure 2a). Sanger sequencing showed that the RPA products of F2/R2 had peaks of ‘C’ and ‘T’ at c.840, while the RPA products of F3/R3 had only one peak of ‘C’ at c.840 (Figure 2b), indicating that the RPA primer pairs F3/R3 were preferable for amplifying *SMN1* specifically.

### 3.3. Establishment of the SMA-Cas12a-Strip Assay

**Figure 3:**
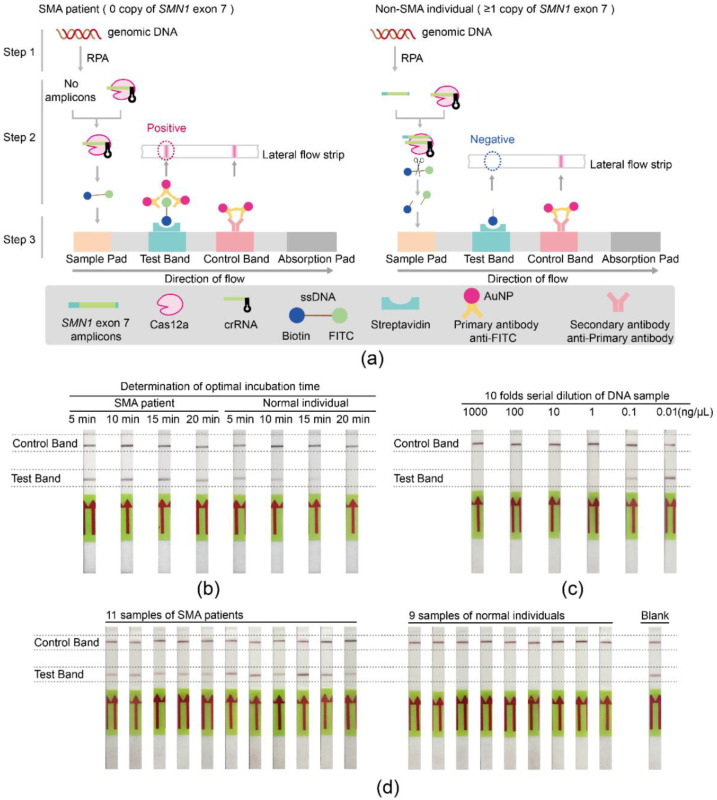
Schematic diagram and validation of the SMA-Cas12a-strip assay. (**a**) For SMA patients with the homozygous deletion of *SMN1* exon 7, no *SMN1* exon 7 amplicons were amplified by RPA (Step 1). Without activation by the amplicons, Cas12a could not cleave the ssDNA probe (Step 2). The products of the Cas12a cleavage reaction were mixed with the buffer and then added to the sample pad of the lateral flow strip. The intact ssDNA probe migrated toward the absorption pad and was captured by streptavidin, which was immobilized at the test band. Subsequently, the FITC was recognized and bound by the primary antibody that was conjugated with gold nanoparticles (AuNP). The aggregation of AuNP at the test band generated a red line, which indicated the integrity of the probe, the inactivation of Cas12a, and the homozygous deletion of *SMN1* exon 7. The excessive primary antibody coupled with AuNP continued migrating toward the absorption pad and was captured by the secondary antibody that was immobilized at the control band. The aggregation of AuNP at the test band generated another red line, which indicated the success of the assay and the validity of the result (Step 3). On the contrary, there was at least one copy of *SMN1* exon 7 in non-SMA individuals, which could be amplified by RPA (Step 1). Amplicons triggered Cas12a and activated Cas12a degraded the ssDNA probe through the collateral cleavage activity, leading to the separation of FITC and biotin (Step 2). When the products of the Cas12a cleavage reaction were mixed with the buffer and added to the sample pad, the biotin terminal of the ssDNA probe was captured at the test band by streptavidin, but the FITC terminal was not retained at the test band. Thus, there was no aggregation of AuNP and no red line at the test band, which indicated the degradation of the probe, the activation of Cas12a, and the existence of *SMN1* exon 7. Primary antibody and AuNP aggregated at the test band and generated a red line. Briefly, only one red line at the control bands was considered as negative, indicating the existence of *SMN1* exon 7. (**b**) The optimal incubation time of RPA products with the Cas12a cleavage reaction system. (**c**) The minimum detectable concentration of genomic DNA with the SMA-Cas12a-strip assay. (**d**) Validation of the SMA-Cas12a-strip assay with DNA from 11 SMA patients, 9 normal controls, and a blank of water.

To improve the simplicity and convenience of detection for SMA, the SMA-Cas12a-strip assay was established. The fluorescence probe was replaced with the ssDNA probe tagged with FITC and biotin at each end, and the method of the reading signal was changed from the fluorometer to the lateral flow strip. The principle of the SMA-Cas12a-strip assay is illustrated in Figure 3a. In Step 1, RPA was applied to amplify *SMN1* exon 7. In Step 2, the amplification products could be specifically recognized by Cas12a and subsequently trigger Cas12a to cleave the FITC-Biotin ssDNA probe through the collateral cleavage activity. In Step 3, the signal of whether the probe had been cleaved could be read by naked eyes.

The optimal incubation time of RPA products with Cas12a was determined by trying four time points (5, 10, 15, and 20 min). For normal individuals, the signals in the test bands gradually faded with the extension of the incubation time and completely disappeared when the incubation time was 20 min. For SMA patients, the positive signals in the test bands were obvious and independent of the incubation time. To balance the specificity and turnaround time of the test, the optimal incubation time was selected to be 20 min (Figure 3b).

The minimum detectable concentration of genomic DNA was evaluated by testing serial dilutions of DNA from one normal donor. Negative signals in the test band were evident enough when DNA concentrations were above 1 ng/μL (Figure 3c). Using the molecular weight of the human genome (1.9 × 10^12^ g/mol, ~2.9 Gb) [27,28], this concentration could be converted into approximately 526 aM.

DNA samples from 20 individuals were amplified by RPA with primer pairs F3/R3, and the products of RPA were tested by the SMA-Cas12a-strip assay. Eleven strips showed positive, and nine strips showed negative in the test band (Figure 3d). The results of 20 individuals tested by the SMA-Cas12a-strip assay showed complete concordance with their genotypes.

### 3.4. Analytical Performance of the SMA-Cas12a-Strip Assay

Sensitivity and specificity were evaluated with 168 samples, including 90 SMA patients and 78 non-SMA individuals, and their genotypes of *SMN1* were confirmed by MLPA and qPCR. After being tested by the SMA-Cas12a-strip assay, all 90 SMA patients had a positive signal in the test bands and were considered as true positives. A total of 78 non-SMA individuals had a negative signal in the test bands and were considered as true negatives (Figure S1). Through the evaluation, the sensitivity and specificity of the SMA-Cas12a-strip assay were both 100% (Table 1).

### 3.5. Extensible Utility of the Cas12a-Strip Assay

**Figure 4:**
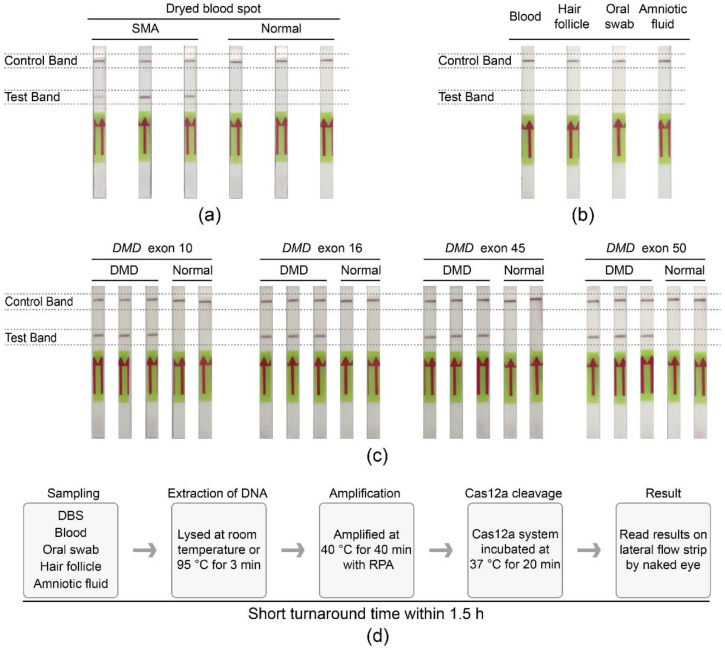
Extensible utility of the Cas12a-strip assay. (**a**) The SMA-Cas12a-strip assay was compatible with DBS samples from newborn screening. (**b**) DNA extracted from blood, hair follicles, oral swabs, and amniotic fluid was also suitable for the SMA-Cas12a-strip assay. (**c**) The Cas12a-strip assay was feasible to detect DMD. (**d**) Process of the SMA-Cas12a-strip assay.

To improve the convenience of the SMA-Cas12a-strip assay, a simple and rapid DNA extraction procedure was integrated with the assay. Three dried blood spots (DBS) samples of SMA patients were tested as positive, and 3 DBS samples from normal newborns were negative (Figure 4a). Moreover, specimens of blood, hair follicles, oral swabs, and amniotic fluid were also suitable for the assay, and the results were consistent with the genotypes of donors (Figure 4b).

To explore the expandability of the assay in other genetic diseases, DNA samples from Duchenne muscular dystrophy (DMD) patients and non-DMD individuals were tested with the DMD-Cas12a-strip assay. All DMD patients manifested positive signals in concordance with the deletions in the dystrophin gene (*DMD*), whereas normal individuals showed negative signals in the test bands (Figure 4c).

The whole process of the SMA-Cas12a-strip assay, including sampling, DNA extraction, RPA, incubation of the Cas12a cleavage reaction system, and the reading of results by naked eyes, could be accomplished within 1.5 h with little auxiliary equipment (Figure 4d).

## 4. Discussion

SMA is one of the most severe and common genetic diseases leading to infant death, and several techniques have been developed to detect the homozygous deletion of *SMN1* exon 7. MLPA is considered the gold standard technique for diagnosis of SMA but has a long turnaround time, a high cost, and a high requirement for DNA samples [13]. qPCR, DHPLC, and HRM hold better application potential in screening for SMA, which has been demonstrated to be feasible and cost-effective by pilot screening [15,16,29]. dPCR could provide more comprehensive and exact results about copy numbers of *SMN1* and *SMN2* [17,18]. However, all these methods need experienced technicians to conduct tests on special instruments and to process data with professional software. The scarcity of these conditions is bound to impede their application in low-resource regions. Compared with the techniques mentioned above, the SMA-Cas12a-strip assay has more accessibility, has fewer limitations, and could be taken as a screening tool for SMA.

The emergence of CRISPR/Cas nucleic acid detection heralds a new era of genetic testing. Recent studies leveraging the collateral cleavage activity of Cas13 and Cas12 mainly focus on detecting the nucleic acid of pathogens, including the Zika virus, Dengue virus, Hantavirus, human papillomavirus, African swine fever viruses, and 2019-nCov [30,31,32,33,34,35], but there has been little focus on disease-causing mutations in human genetic diseases. Another novel method combining the gene targeting capacity of Cas9 with the sensitive detection ability of graphene-based field-effect transistors can detect deletions of *DMD* without pre-amplification [27]. However, it is uncertain whether this method could detect the homozygous deletion of *SMN1* because of the interference of *SMN2* in the human genome.

SMN1 crRNA-wt was able to discriminate SMA patients from normal individuals to some extent, but nonspecific activation of the Cas12a/SMN1 crRNA-wt complex by *SMN2* in SMA patients still occurs. In order to avoid the interference of *SMN2* and improve the sensitivity to detect the homozygous deletion of *SMN1* exon 7, an artificial mismatch adjacent to c.840 was introduced into SMN1 crRNA-mut for a more discernable difference between SMA patients and normal individuals.

Amplification was necessary for the assay. Cas12a cannot be triggered to generate an evident fluorescence without PCR, even with a high concentration of DNA (1000 ng/μL). Compared with PCR, RPA is more suitable for the SMA-Cas12a-strip assay, owning to the lower requirements for DNA samples and reaction conditions (incubation at 35–42 °C) [36]. By means of RPA, the SMA-Cas12a-strip assay could detect the DNA target at concentrations as low as 526 aM, which ensured that a small amount of DNA from DBS and other specimens could be used for detection. The analytical performance of the SMA-Cas12a-strip assay was assessed by 168 clinical samples, indicating a sensitivity of 100% and a specificity of 100%. However, the sample size for validating the sensitivity and specificity was relatively small, and larger sample size is needed to verify the analytical performance.

The SMA-Cas12a-strip assay had a short turnaround time, a low cost, and a low requirement for instruments. The whole test process could be accomplished within 1.5 h, and the technical cost was anticipated to be $0.76 USD per test. The only necessary reaction condition was incubation, and the requirement for the accuracy of temperature was less than that of PCR, which could be easily realized by a water bath or a dry bath incubator. It is possible to integrate the test process into a single and portable device that contains separate chambers for each reaction and channels for liquid transfer among chambers. Moreover, the reaction temperatures of RPA and Cas12a cleavage are close. If a reaction system compatible with the two reactions could be found, the RPA and Cas12a cleavage could be combined in one reaction, and the test process could be further simplified.

DNA extracted from DBS, hair follicles, and oral swabs was also proved to be suitable for the SMA-Cas12a-strip assay, which provided convenience with sampling and DNA extraction. Moreover, we modified the Cas12a-strip assay to detect DMD as a proof-of-concept for its expandable application in detecting other genetic diseases.

It is worth noting that the SMA-Cas12a-strip assay could qualitatively detect the homozygous deletion of *SMN1* exon 7, which accounts for more than 95% of SMA patients, but the copy numbers of *SMN1* and *SMN2* and the rare mutations in *SMN1* are beyond this assay’s capacity.

## 5. Conclusions

In this study, Cas12a based nucleic acid detection, the isothermal amplification of RPA, and the lateral flow strip were integrated to establish an SMA-Cas12a-strip assay. Due to its simplicity and low cost, the SMA-Cas12a-strip assay holds great promise in the application, even in underdeveloped countries or low-resource areas.

## Figures and Tables

**Table 1:** The analytical performance of the SMA-Cas12a-strip assay.

		Confirmed by MLPA ^1^/qPCR ^2^	
		SMA Patients	Non-SMA Individuals
Tested by the SMA-Cas12a-Strip Assay	Positive	90 (TP) ^3^	0 (FP) ^4^
	Negative	0 (FN) ^5^	78 (TN) ^6^
		Sensitivity ^7^: 100%	Specificity ^8^: 100%

^1^ MLPA: multiplex ligation-dependent probe amplification. ^2^ qPCR: quantitative PCR. ^3^ TP: true positive. ^4^ FP: false positive. ^5^ FN: false negative. ^6^ TN: true negative. ^7^ Sensitivity = TP/(TP + FN). ^8^ Specificity = TN/(TN + FP).

## Supplementary Materials

The following are available online at https://www.mdpi.com/article/10.3390/bios11050154/s1, Table S1: Information of samples used in the SMA assay. Table S2: Information of samples used in the DMD assay. Table S3: Detailed sequence of primers, crRNAs, and probes. Figure S1: Assessment of sensitivity and specificity of the SMA-Cas12a-strip with 168 clinical samples.
