# Supplementary material for: Cas12a and Lateral Flow Strip-Based Test for Rapid and Ultrasensitive Detection of Spinal Muscular Atrophy
Source: Biosensors (Basel). 2021 May 14;11(5):154. doi: 10.3390/bios11050154 (PMC8153588; doi:10.3390/bios11050154)
Supplement: Supplementary file 1 [file biosensors-11-00154-s001.zip › biosensors-1193064-supplementary.pdf]

Table S1. Information of samples used in SMA assay.

Sample source	Cohort	Genotype of *SMN1* exon 7	Number of samples
Residual DNA	SMA patients	Homozygous deletion	101
Residual DNA	SMA carriers	Heterozygous deletion	9
Residual DNA	Non-SMA individuals	No deletion	78
DBS	SMA patients	Homozygous deletion	3
DBS	Non-SMA neonates	No deletion	3
Oral swab	Non-SMA donor	No deletion	1
Hair follicle	Non-SMA donor	No deletion	1
Blood	Non-SMA donor	No deletion	1
Amniotic fluid	Non-SMA fetus	No deletion	1

Table S2. Information of samples used in DMD assay.

Samples	Genotype of *DMD*	Target exons to assay
DMD patient 1	Deletion of exon 3-17	Exon 10 and 16
DMD patient 2	Deletion of exon 8-42	Exon 10 and 16
DMD patient 3	Deletion of exon 3-43	Exon 10 and 16
DMD patient 4	Deletion of exon 45-52	Exon 45 and 50
DMD patient 5	Deletion of exon 45-54	Exon 45 and 50
DMD patient 6	Deletion of exon 45	Exon 45
DMD patient 7	Deletion of exon 48-52	Exon 50
Non-DMD donor 1	No deletion	Exon 10 and 16
Non-DMD donor 2	No deletion	Exon 10
Non-DMD donor 3	No deletion	Exon 16
Non-DMD donor 4	No deletion	Exon 45 and 50
Non-DMD donor 5	No deletion	Exon 45 and 50

Table S3. Detailed sequence of primers, crRNAs and probes.

Target gene	Category	Sequence
SMN1 (Exon 7)	PCR primer F1	5'-AAAATGTCTTGTGAAACAAAATGC-3'
	PCR primer R1	5'-CACTTTCATAATGCTGGCAGAC-3'
	RPA primer F2	5'-AACTGCAGCCTAATAATTGTTTTCTTTGGGAT-3'
	RPA primer R2	5'-CAAACCATAAAGTTTTACAAAAGTAAGATTCAC-3'
	RPA primer F3	5'-ATATAAAGCTATCTATATATAGCTATCTATG-3'
	RPA primer R3	5'-TATTGATTGTTTTACATTAACCTTTCAACTTTT-3'
	crRNA-wt	5'-UAAUUUCUACUAAGUGUAGAUUCUGAAACCC UGUAAGGAAAAUA-3'
	crRNA-mut	5'-UAAUUUCUACUAAGUGUAGAUUCUGGAACCC UGUAAGGAAAAUA-3'
DMD (Exon 10)	RPA primer F	5'-ATCTGCTGTTCCTGTGTTTGATAATGCCAGTG-3'
	RPA primer R	5'-GAGTAATTGAGGAAAAAGGATGACTTGCCATT-3'
	crRNA	5'-UAAUUUCUACUAAGUGUAGAUUGCUGAGGAC ACAUUGCAAGCAC-3'
DMD (Exon 16)	RPA primer F	5'-ATTTTTCTGAACTTTTGATCCTTTGCGGGCAC-3'
	RPA primer R	5'-GCATGATAATTGGTATCACTAACCTGTGCTGT-3'
	crRNA	5'-UAAUUUCUACUAAGUGUAGAUUAGAUCCGCU UUUAAAACCUGUU-3'
DMD (Exon 45)	RPA primer F	5'-GTACAACTGCATGTGGTAGCACACTGTTTAAT-3'
	RPA primer R	5'-TTCTTTAATGTTAGTGCCTTTCACCCTGCTTA-3'
	crRNA	5'-UAAUUUCUACUAAGUGUAGAUCCGCUGCCCA AUGCCAUCCUGGA-3'
DMD (Exon 50)	RPA primer F	5'-TGATAAATATTTGTAGGGTGGTTGGCTAAAAT-3'
	RPA primer R	5'-TTCTCTCTCACCCAGTCATCACTTCATAGTTG-3'
	crRNA	5'-UAAUUUCUACUAAGUGUAGAUCCGCCUUCCA CUCAGAGCUCAGA-3'
No target	Fluorescence probe	5'-CY5/CCTGTTCTTTAGCTATCCGTG/BHQ-X-3'
	FITC-Biotin probe	5'-FITC/ACACACACACACACACACAC/Biotin-3'

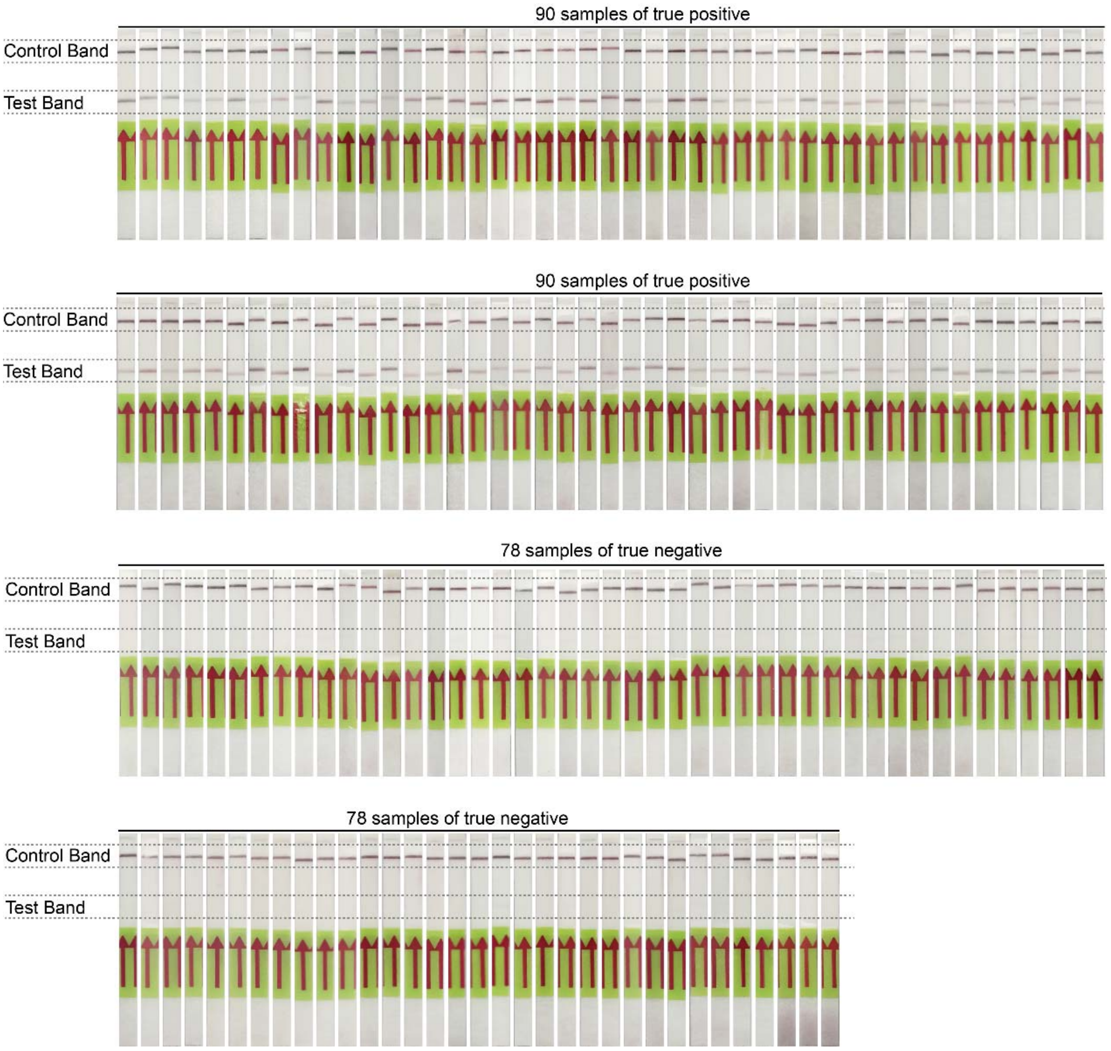

Figure S1. Assessment of sensitivity and specificity of the SMA-Cas12a-strip with 168 clinical samples.
